# Phylogeographic Evidence for a Link of Species Divergence of *Ephedra* in the Qinghai-Tibetan Plateau and Adjacent Regions to the Miocene Asian Aridification

**DOI:** 10.1371/journal.pone.0056243

**Published:** 2013-02-13

**Authors:** Ai-Li Qin, Ming-Ming Wang, Yu-Zhi Cun, Fu-Sheng Yang, Shan-Shan Wang, Jin-Hua Ran, Xiao-Quan Wang

**Affiliations:** 1 State Key Laboratory of Systematic and Evolutionary Botany, Institute of Botany, The Chinese Academy of Sciences, Beijing, People's Republic of China; 2 University of Chinese Academy of Sciences, Beijing, People's Republic of China; Wuhan Botanical Garden, Chinese Academy of Sciences - Wuhan, China

## Abstract

The Qinghai-Tibetan Plateau (QTP) has become one of the hotspots for phylogeographical studies due to its high species diversity. However, most previous studies have focused on the effects of the Quaternary glaciations on phylogeographical structures and the locations of glacial refugia, and little is known about the effects of the aridization of interior Asia on plant population structure and speciation. Here the chloroplast DNA (cpDNA) *trn*T-*trn*F and *trn*S-*trn*fM sequences were used to investigate the differentiation and phylogeographical history of 14 *Ephedra* species from the QTP and northern China, based on a sampling of 107 populations. The phylogeographical analysis, together with phylogenetic reconstruction based on combined four cpDNA fragments (*rbc*L, *rpl*16, *rps*4, and *trn*S-*trn*fM), supports three main lineages (eastern QTP, southern QTP, and northern China) of these *Ephedra* species. Divergence of each lineage could be dated to the Middle or Late Miocene, and was very likely linked to the uplift of the QTP and the Asian aridification, given the high drought and/or cold tolerance of *Ephedra*. Most of the *Ephedra* species had low intraspecific variation and lacked a strong phylogeographical structure, which could be partially attributed to clonal reproduction and a relatively recent origin. In addition, ten of the detected 25 cpDNA haplotypes are shared among species, suggesting that a wide sampling of species is helpful to investigate the origin of observed haplotypes and make reliable phylogeographical inference. Moreover, the systematic positions of some *Ephedra* species are discussed.

## Introduction

The Late Cenozoic uplift of the Qinghai-Tibetan Plateau (QTP), the highest and largest plateau in the world with a mean elevation of 4500 m and an area of 2.5×10^6^ km^2^ [1], resulted in Asian aridification [2], [3], and promoted the development of a rich biodiversity in the southern and southeastern QTP, where three world biodiversity hotspots were recognized [4]. For instance, the Himalaya-Hengduan Mountains region harbors over 20,000 species of vascular plants, which represent the richest alpine flora on the earth with a high percentage of endemic species [5], [6]. However, many fewer plants are distributed in the vast QTP platform due to extreme cold and arid environments. The mechanisms underlying species diversification in the QTP have fascinated biologists for a long time (e.g., [7]–[15]). Do the congeneric plant species in the QTP have similar evolutionary and biogeographic history? Did they differ in response to historical climatic changes?

In recent years, the QTP has become one of the hotspots for plant phylogeographical studies, such as first on *Pinus densata* [16], [17], and then on diverse subalpine and alpine plants (e.g., [18]–[21]). Most of these studies suggested postglacial/interglacial plant colonization/recolonization of the QTP platform and the Himalayas from the adjacent lower-elevation regions, especially from the Hengduan Mountains [18]–[22], or glacial in situ survival in microrefugia on the QTP platform [12], [23], [24]. While most previous studies have focused on the effects of the Quaternary glaciations on phylogeographical structures and the locations of glacial refugia, little is known about the effects of the aridization of interior Asia, partially driven by the QTP uplift and global cooling, on plant population structure and speciation. In addition, the Central Asian plants were very rarely investigated in these studies.

On the other hand, although phylogeographical studies have been conducted in diverse plant groups and in many geographical regions [25]–[29], most of them sampled a single or a couple of closely related species at population level. This makes it difficult to investigate the origin of the haplotypes detected in the studied group. For instance, a haplotype of a species or population, whether rare or common, might be newly evolved or inherited from a common ancestor [10], [20], and could also be obtained by interspecific gene flow. When the evolutionary history of the haplotype and its distribution in other species are unknown, an incorrect phylogeographical inference could be made, especially for studies of groups with high dispersal ability or a complicated evolutionary history.

The genus *Ephedra* is mainly shrubs and comprises about 50 species [30], most of which are extremely drought and/or cold tolerant. Approximately sixteen *Ephedra* species occur in the QTP and adjacent regions [31]–[33], and all of them could have originated in the Late Cenozoic by adaptive radiation and are relatively closely related [30], [34], [35], making them ideally suited for the investigation of topographic and climatic effects on plant population dynamics and speciation. For example, *Ephedra saxatilis*, a species mainly distributed in the Himalayas, has red and fleshy cone bracts adapted to animal dispersal. In contrast, *E. przewalskii* that is widely distributed in the deserts of Central Asia has winged cone bracts suitable for wind dispersal.

In the present study, we use chloroplast DNA (cpDNA) *trn*T-*trn*F and *trn*S-*trn*fM sequences to investigate the differentiation and phylogeographical history of the *Ephedra* species distributed in the QTP and adjacent regions, based on a wide population sampling. The questions to be addressed include: (1) Has the species diversification of *Ephedra* been driven by the QTP uplift and the Asian aridization? (2) Are the phylogeographical patterns of the *Ephedra* species similar to those revealed in other QTP plants? (3) How did the *Ephedra* populations respond to the Quaternary climatic changes? (4) How important is species sampling in phylogeographical studies?

## Materials and Methods

### Ethics statement

No specific permits were required for the described field studies.

### Sampling

**Figure 1:**
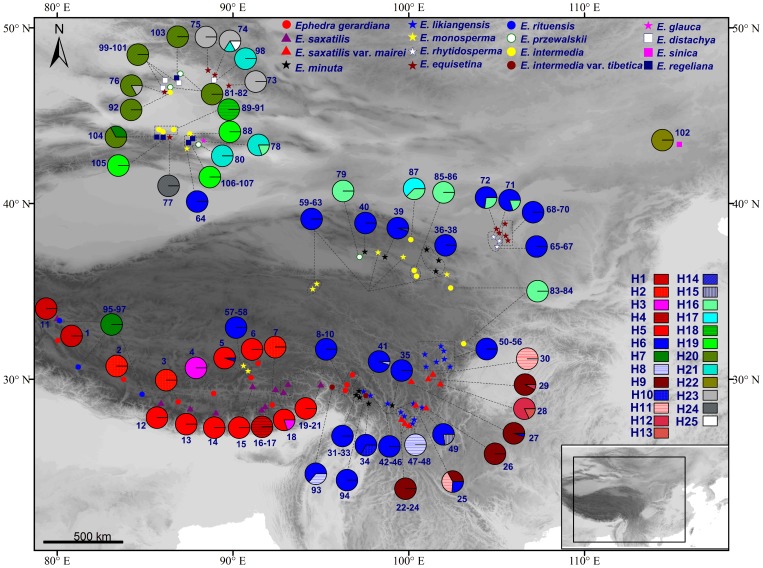
Sampling locations and distribution frequencies of the cpDNA haplotypes of 14 *Ephedra* species. Population numbers correspond to those in Table 1.

Except *E. fedtschenkoae* and *E. lomatolepis* that are difficult to access, all the other 14 *Ephedra* species distributed in the QTP and adjacent regions were sampled for the phylogeographical study based on cpDNA *trn*T-*trn*F and *trn*S-*trn*fM sequences (Fig. 1). Young branchlets were collected from 107 populations (totaling 1435 individuals), most of which were represented by 5–29 individuals that were at least 50 m apart from each other. For the species mainly distributed in the QTP, the sample size is larger than 12 for most populations. The population codes, sample sizes, and geographical coordinates are shown in Table S1. Also, one individual of the Mediterranean *E. nebrodensis* was sampled as outgroup based on the results of previous phylogenetic analyses [30], [35], [36]. To better understand the phylogeographical patterns of the 14 *Ephedra* species, their evolutionary relationships were also reconstructed from sequence analysis of combined four cpDNA fragments (*rbc*L, *rpl*16, *rps*4, and *trn*S-*trn*fM), in which most species were represented by 2–4 individuals and the DNA sequences of other congeneric species available in GenBank were included. In total, 37 species were sampled in the combined cpDNA analysis (Table S2). *E. saxatilis* var. *mairei* and *E. intermedia* var. *tibetica* were considered as two independent taxa in all analyses due to their unique phylogenetic positions (see discussion). The species status of *P. glauca* was recognized in a recent revision of the genus *Ephedra* from China [32], and thus was followed in our study.

### DNA extraction, PCR amplification, and sequencing

Total genomic DNA was isolated from silica gel-dried young branchlets using the modified CTAB method [37]. The cpDNA *trn*T-*trn*F and *trn*S-*trn*fM regions were amplified with the primer pairs *trn*T (5′-CATTACAAATGCGAT GCTCT-3′) and *trn*F (5′-ATTTGAACTGGTGACACGAG-3′) [38], *trn*S (5′-GAGAGAGAGGGATTCG AACC-3′) and *trn*fM (5′-CATAACCTTGAGGTCACGGG-3′) [39], respectively. Other primers for the amplification of cpDNA are shown in Table S3. The polymerase chain reaction (PCR) was conducted in a Mastercycler (Eppendorf, Hamburg, Germany) or a Tgradient Thermocycler (Biometra) in a volume of 25 µL, containing 5–50 ng plant DNA, 6.25 pmol of each primer, 200 µmol/L of each dNTP, and 0.75 unit of Taq DNA polymerase (TakaRa Biotech Co., Dalian, China). PCR cycles were as follows: 4 min at 94°C, three cycles of 2 min at 94°C, 30 s at 52°C, and 1–1.5 min at 72°C, followed by 33 cycles of 30 s at 94°C, 30 s at 54°C, and 1–1.5 min at 72°C, with a final extension step of 10 min at 72°C.

The PCR products were purified using a Gel Band Purification Kit (Amersham Biosciences, Buckinghamshire, UK) or PEG 8000, and then were directly sequenced using ABIPrism BigDye Terminator Cycle Sequencing Ready Reaction Kit or DYEnamic Energy Transfer (ET) Terminator Reagent Premix Kit. After precipitation with 95% EtOH and 3M NaAc (pH 5.3), the sequencing products were separated on an ABI PRISM 3730xl analyzer (Applied Biosystems) or a MegaBACE 1000 automatic sequencer (Amersham Biosciences). The sequences reported in this study are deposited in GenBank under accession numbers KC222975–KC223020 (*rbc*L), KC223021–KC223066 (*rpL*16), KC223067–KC223112 (*rps*4), KC223113–KC223158 and KC407804–KC407829 (*trn*S-*trn*fM), and KC407778–KC407803 (*trn*T-*trn*F).

### Data analyses

The DNA sequences were aligned using the program Clustal X [40] and manually adjusted in BioEdit v. 7.0.9 [41]. Arlequin 3.11 [42] was used to estimate the molecular diversity indices, including the number of segregating sites (*S*), number of haplotypes (*N_h_*), haplotype diversity (*H*_d_) and nucleotide diversity (*π*), for each population and species. The haplotype richness (*A*) was calculated by dividing the number of haplotypes by the number of sampled individuals (*N*). The program PERMUT [43] was used to calculate average gene diversity within populations (*H_S_*), total gene diversity (*H_T_*), and two measures of population differentiation, i.e., *G_ST_* [44] and *N_ST_* (equivalent coefficient taking into account sequence similarities between haplotypes). An analysis of molecular variance (AMOVA) [42] and a Mantel test [45] were performed in Arlequin 3.11 to partition variation within and among populations and to assess the correlation between genetic and geographic distances, respectively. Also, the DNA divergence between populations (*F_ST_*, [42]) was measured, and the significance was tested using 10,000 permutations. A network of the cpDNA haplotypes (chlorotypes) was constructed using TCS 1.21 [46], with a default parsimony connection limit of 95% and each insertion/deletion (indel) treated as a single mutation event.

The evolutionary relationships of the chlorotypes were also reconstructed with maximum parsimony (MP), maximum likelihood (ML) and Bayesian inference (BI), using PAUP*4.0b10 [47], PhyML 3.0 [48], and MrBayes 3.1.2 [49], respectively. All phylogenetically informative gaps (indels) were coded as single mutation events in the final alignment. In the MP analysis, all characters were treated as unordered and equally weighted, and a heuristic search was implemented with 1000 random addition sequence replicates, tree-bisection-reconnection (TBR) branch swapping and MULTREES on. To examine the robustness of clades in the most parsimonious trees, a bootstrap analysis was conducted with 1000 replicates using the same heuristic search settings as described above. In the ML analysis, we chose the K81uf+I model, which was determined to be the best-fit model for the cpDNA dataset by the Akaike Information Criterion (AIC) implemented in Modeltest 3.06 [50]. The Bayesian analysis used the best-fit model HKY+I determined by AIC in MrModeltest 2.3 [51] and random starting trees. One cold and three incrementally heated Markov chain Monte Carlo (MCMC) chains were run for 1,000,000 generations each, sampling one tree per 100 generations with the first 300 samples discarded as burn-in. The trees sampled after generation 30,000 were used for phylogenetic inference. In the phylogenetic reconstruction of the *Ephedra* species based on the combined four chloroplast genes, the ML and BI analyses were performed with the best-fit models TVM+I+G and GTR+R+G, respectively.

To detect whether historical population expansion events occurred in the *Ephedra* species, mismatch distributions were calculated using the program Arlequin 3.11 [42]. Based on 1000 parametric bootstrap replicates, an expected distribution was generated under a model of sudden demographic expansion [52]. The sum-of-squared deviations (SSD) between observed and expected mismatch distributions were calculated, with the *P*-values representing the proportion of simulations producing a larger SSD than the observed SSD.

Divergence times of the chlorotypes were estimated by molecular clock analysis. The rate constancy among lineages was evaluated by a likelihood ratio test (LRT), which compared log likelihood ratios of the chosen model with and without an enforced molecular clock [53]. Significance was assessed by comparing two times the difference in log likelihood to a chi-square distribution, with the degree of freedom equal to the number of taxa minus two. Since the clock assumption was rejected (*δ* = 41.5215, df = 24, *P*<0.05), divergence times were estimated with BEAST v1.7.2 [54], using the HKY+I+G substitution model, an uncorrelated lognormal relaxed clock model, the Yule model of speciation and a user-specified starting tree (ML tree). The root age was set to a median value of 29.56 Ma based on the time of the most recent common ancestor of ‘Core *Ephedra*’ [30] and in consideration of the fact that the New World *Ephedra* species were nested within the Asian clade in some previously published phylogenies [30], [55], [56] and the combined cpDNA phylogeny constructed in the present study, and minimally 23.03 Ma according to the fossil record (pollen) of *Ephedra* in the Late Oligocene sediments of the QTP and neighboring regions [57]–[59]. The MCMC analysis was run for 10,000,000 generations to estimate the mean posterior divergence times with standard deviations based on the variance-covariance matrix, a sampling frequency of every 1000 generations and a burn in of 1000. The program TRACER v.1.5 [60] was used to check convergence of chains to the stationary distribution. The MCMC output was analyzed with TreeAnnotator v1.5.4, and the chronological tree was visualized by FigTree v1.3.1.

## Results

### Distributions and evolutionary relationships of cpDNA haplotypes

In the phylogeographical study, we obtained *trn*T-*trn*F and *trn*S-*trn*fM sequences from all of the 1435 individuals surveyed. The alignment of the combined two cpDNA fragments was 1177 bp in length, including 20 nucleotide substitutions and 8 indels (1–27 bp in size) that were used to designate 25 haplotypes (H1–H25) (Tables 1, 2). *Ephedra equisetina* had the most haplotypes (7), although the total sample size (*N* = 64) for this species is not very large. The number of detected haplotypes was five in *E. gerardiana* (*N* = 196), *E. saxatilis* var. *mairei* (*N* = 183) and *E. intermedia* (*N* = 96), four in *E. saxatilis* (*N* = 197), three in *E. minuta* (*N* = 158), *E. likiangensis* (*N* = 183), *E. przewalskii* (*N* = 22) and *E. regeliana* (*N* = 27), and two in *E. intermedia* var. *tibetica* (*N* = 19), *E. glauca* (*N* = 5) and *E. distachya* (*N* = 15), respectively. No intraspecific variation was found in *E. rhytidosperma* (*N* = 17), *E. monosperma* (*N* = 96), *E. rituensis* (*N* = 66), and *E. sinica* (*N* = 13) (Fig. 1; Table 1). The haplotype H6 was the most widely distributed, and was shared by eight species (*E. equisetina*, *E. gerardiana*, *E. intermedia* var. *tibetica*, *E. likiangensis*, *E. minuta*, *E. monosperma*, *E. rhytidosperma* and *E. saxatilis* var. *mairei*). Also, each of the nine haplotypes (H1, H3, H5, H7, H8, H16, H19–H21) was shared by two or four species. The rest haplotypes were species-specific (Fig. 1; Table S4).

**Table 1:** The cpDNA *trn*T-*trn*F+*trn*S-*trn*fM haplotypes detected in the sampled populations of 14 *Ephedra* species.

Species	Population No.	*N*	Haplotypes (Individuals)	Species	Population No.	*N*	Haplotypes (Individuals)
*Ephedra gerardiana*	1	24	H1 (24)		55	10	H6 (10)
	2	27	H2 (27)		56	22	H6 (22)
	3	16	H2 (16)	*E. monosperma*	57	14	H6 (14)
	4	24	H3 (24)		58	8	H6 (8)
	5	23	H5 (22), H6 (1)		59	16	H6 (16)
	6	25	H5 (25)		60	23	H6 (23)
	7	3	H2 (3)		61	17	H6 (17)
	8	25	H6 (25)		62	6	H6 (6)
	9	12	H6 (12)		63	6	H6 (6)
	10	17	H6 (17)		64	6	H6 (6)
*E. saxatilis*	11	14	H1 (14)	*E. rhytidosperma*	65	5	H6 (5)
	12	24	H5 (24)		66	6	H6 (6)
	13	6	H5 (6)		67	6	H6 (6)
	14	25	H5 (25)	*E. equisetina*	68	6	H6 (6)
	15	29	H5 (29)		69	6	H6 (6)
	16	6	H4 (6)		70	6	H6 (6)
	17	3	H4 (3)		71	6	H6 (5), H16 (1)
	18	25	H3 (5), H5 (20)		72	5	H6 (4), H16 (1)
	19	29	H5 (29)		73	10	H23 (10)
	20	16	H5 (16)		74	6	H21 (1), H23 (4), H25 (1)
	21	20	H5 (20)		75	7	H23 (7)
*E. saxatilis* var. *mairei*	22	24	H9 (24)		76	6	H20 (5), H23 (1)
	23	29	H9 (29)		77	6	H24 (6)
	24	12	H9 (12)	*E. glauca*	78	5	H16 (1), H21 (4)
	25	12	H6 (3), H9 (4), H11 (5)	*E. przewalskii*	79	5	H16 (5)
	26	17	H9 (17)		80	5	H21 (5)
	27	23	H6 (1), H9 (22)		81	6	H20 (6)
	28	28	H12 (23), H13 (5)		82	6	H20 (6)
	29	26	H9 (24), H12 (2)	*E. intermedia*	83	5	H16 (5)
	30	12	H11 (12)		84	5	H16 (5)
*E. minuta*	31	25	H6 (25)		85	27	H16 (27)
	32	17	H6 (17)		86	22	H16 (22)
	33	22	H6 (22)		87	16	H16 (6), H17 (10)
	34	3	H6 (2), H10 (1)		88	2	H19 (2)
	35	6	H6 (6)		89	5	H18 (5)
	36	20	H6 (20)		90	6	H18 (6)
	37	3	H6 (3)		91	6	H18 (6)
	38	26	H6 (26)		92	2	H20 (2)
	39	20	H6 (19), H14 (1)	*E. intermedia* var. *tibetica*	93	14	H6 (9), H8 (5)
	40	16	H6 (16)		94	5	H6 (5)
*E. likiangensis*	41	22	H6 (21), H8 (1)	*E. rituensis*	95	12	H7 (12)
	42	5	H6 (5)		96	30	H7 (30)
	43	24	H6 (24)		97	24	H7 (24)
	44	13	H6 (13)	*E. distachya*	98	3	H21 (3)
	45	25	H6 (25)		99	5	H20 (5)
	46	22	H6 (22)		100	4	H20 (4)
	47	25	H8 (25)		101	3	H20 (3)
	48	4	H8 (4)	*E. sinica*	102	13	H22 (13)
	49	13	H6 (10), H15 (3)	*E. regeliana*	103	6	H20 (6)
	50	21	H6 (21)		104	6	H7 (2), H20 (4)
	51	11	H6 (11)		105	5	H19 (5)
	52	5	H6 (5)		106	5	H19 (5)
	53	23	H6 (23)		107	5	H19 (5)
	54	16	H6 (16)	**All species**	**Total**	**1435**	

*N*, number of sampled individuals.

**Table 2:** The cpDNA haplotypes detected in *Ephedra* species.

Hap.	*trn*T-*trn*F (668 bp)																						*trn*S-*trn*fM (509 bp)											
	34	66	77	100	106	111	180	224	234	298	314	324	333	398	437	489	524	543	615	616	627	666	672	698	944–960	973	991	994–1008	1011	1089–1096	1103	1113–1139	1140–1158	1161
H1	C	A	A	-	G	C	G	A	A	A	C	G	A	T	T	T	C	A	T	A	-	-	G	G	-	T	T	§	A	□	T	-	-	A
H2	C	A	A	-	G	C	G	A	A	A	C	G	A	T	T	T	C	A	T	A	-	-	G	G	-	T	G	§	A	□	T	-	-	A
H3	C	A	A	-	G	C	G	A	A	C	C	G	A	T	T	T	C	A	T	A	-	-	G	G	-	T	T	◊	A	□	T	-	-	A
H4	C	A	A	-	G	C	G	A	A	A	C	G	A	T	T	T	C	A	T	A	G	-	G	G	-	T	T	◊	A	□	T	-	-	A
H5	C	A	A	-	G	C	G	A	A	A	C	G	A	T	T	T	C	A	T	A	-	-	G	G	-	T	T	◊	A	□	T	-	-	A
H6	C	A	A	-	G	G	G	A	A	A	A	G	A	T	T	T	C	G	T	A	-	-	G	T	-	T	T	*	G	-	T	-	-	T
H7	C	A	A	A	G	G	G	A	A	A	C	G	A	T	T	T	C	G	T	A	-	-	G	T	#	T	T	◊	G	-	T	-	-	A
H8	C	A	A	-	G	G	G	A	A	A	A	G	A	T	T	T	C	G	T	A	-	-	G	T	-	T	T	Δ	A	-	T	-	-	T
H9	C	A	A	-	G	C	G	A	A	A	C	G	A	T	G	T	C	A	T	A	-	-	G	G	-	T	T	◊	A		T	-	-	A
H10	C	A	A	-	G	G	G	A	A	A	A	G	A	C	T	T	C	G	T	A	-	-	G	T	-	T	T	*	G	-	T	-	-	T
H11	C	A	A	-	G	C	G	A	A	A	C	G	A	T	G	T	C	A	T	A	-	C	G	G	-	T	T	◊	A	□	T	-	-	A
H12	C	A	A	-	G	C	G	A	A	A	C	G	A	T	G	C	C	A	T	A	-	-	G	G	-	T	T	◊	A	□	T	-	-	A
H13	A	A	A	-	G	C	G	A	A	A	C	G	A	T	G	C	C	A	T	A	-	-	G	G	-	T	T	◊	A	□	T	-	-	A
H14	C	A	A	-	G	G	G	A	A	A	A	G	A	T	T	T	C	G	T	A	-	-	G	T	-	T	T	*	G	-	T	†	-	T
H15	C	A	A	-	G	G	G	A	A	A	A	G	C	T	T	T	C	G	T	A	-	-	G	T	-	T	T	*	G	-	T	-	-	T
H16	C	A	A	A	G	G	A	A	A	A	C	G	A	T	T	T	C	G	T	A	-	-	G	T	-	A	T	◊	G	-	T	-	-	A
H17	C	A	A	A	G	G	G	A	A	A	C	G	A	T	T	T	C	G	T	A	-	-	G	T	-	T	T	◊	G	-	T	-	-	A
H18	C	A	A	A	G	G	A	A	A	A	C	G	A	T	T	T	C	G	T	A	-	-	G	T	#	T	T	◊	G	-	T	-	-	A
H19	C	G	A	A	G	G	G	A	A	A	C	G	A	T	T	T	C	G	T	A	-	-	G	T	#	T	T	◊	G	-	T	-	-	A
H20	C	A	T	A	G	G	G	A	A	A	C	G	A	T	T	T	C	G	T	C	-	-	G	T	-	T	T	◊	G	-	T	-	-	A
H21	C	A	T	A	G	G	G	A	A	A	C	G	A	T	T	T	C	G	T	A	-	-	G	T	-	T	T	◊	G	-	T	-	-	A
H22	C	A	A	A	G	G	G	A	A	A	C	G	A	T	T	T	C	G	T	A	-	-	A	T	-	T	T	◊	G	-	T	-	-	A
H23	C	A	A	-	G	G	G	A	A	A	C	G	A	T	T	T	C	G	T	A	-	-	G	T	-	T	T	◊	G	-	C	-	-	A
H24	C	A	A	-	G	G	G	A	A	A	C	G	A	T	T	T	C	G	T	A	-	-	G	T	-	T	G	◊	G	-	C	-	-	A
H25	C	A	A	-	G	G	G	A	A	A	C	G	A	T	T	T	C	G	T	A	-	-	G	T	-	T	T	◊	G	-	C	-	‡	A
H26	C	A	A	-	A	G	G	G	T	A	C	-	A	T	T	T	T	A	G	A	-	-	G	T	-	T	G	◊	G	□	T	-	-	A

#, TCACAAACTTAATAAGT; §, TTGAACGGATTAAAA; ◊, TTGAACGGA------; *, TTAAACGGA------; Δ, ---------------; □, TAAGAATA; †, TATTAAGACAATAAGAATAAGACATAT; ‡, TAATAAGACATAATATGTC. Numbers above lanes indicate positions of the nucleotides in the alignment.

**Figure 2:**
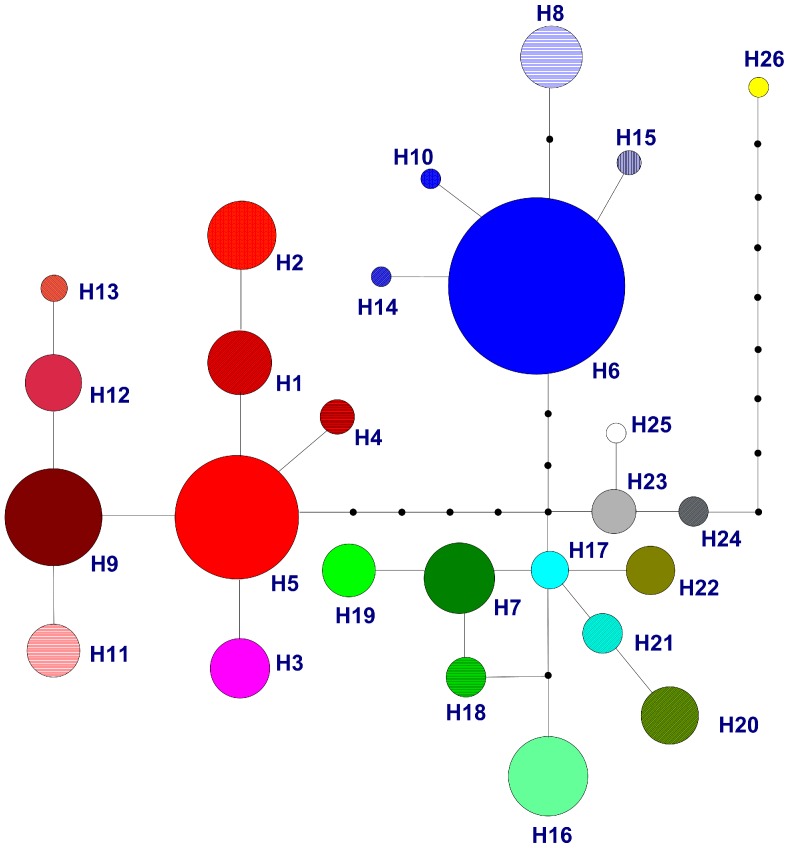
A network of the cpDNA haplotypes constructed by using TCS 1.21. The sizes of the circles in the network are proportional to the observed frequencies of the haplotypes.

**Figure 3:**
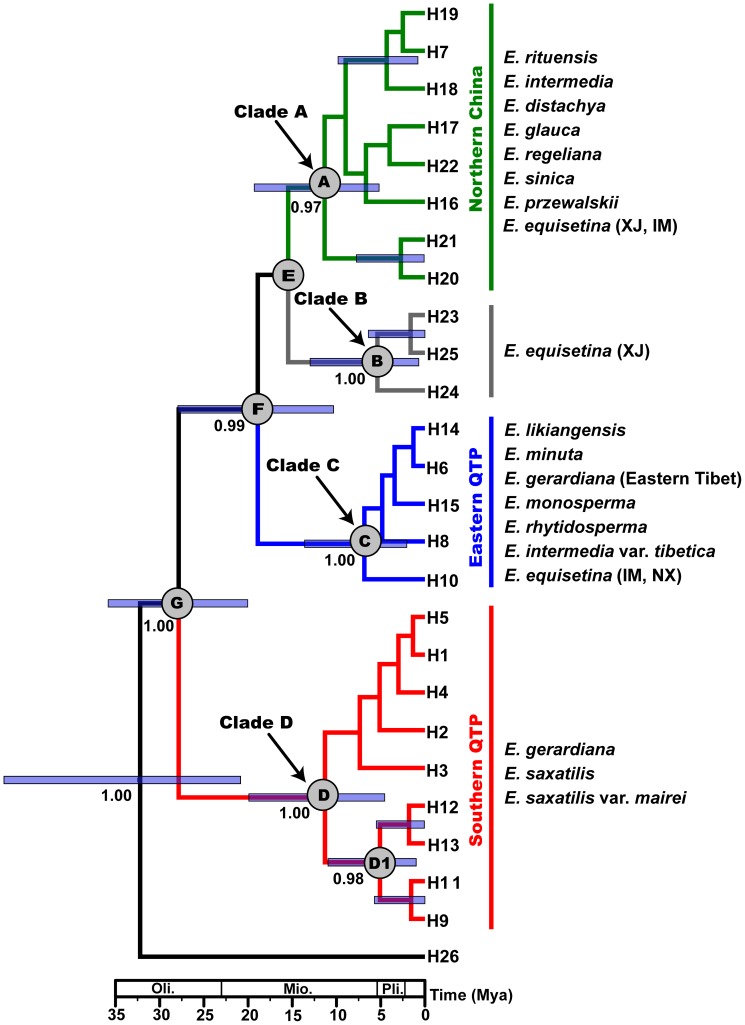
Phylogenetic chronogram of the cpDNA (*trn*T-*trn*F+*trn*S-*trn*fM) haplotypes generated from BEAST. Numbers below the branches indicate the Bayesian posterior probabilities. Median ages of nodes are shown, with horizontal bars indicating the 95% highest posterior density intervals (for details, see Table 3).

Four main lineages were resolved in the network of the cpDNA haplotypes when the sequence of the Mediterranean *E. nebrodensis* (H26) was used as outgroup (Fig. 2). One comprised three haplotypes (H23–25) from *E. equisetina*, a species widely distributed in northern China and most closely related to the outgroup. The other three lineages were mainly distributed in eastern QTP (H6, H8, H10, H14–15), southern QTP (H1–5, H9, H11–13), and northern China (H7, H16–22), respectively. The four lineages were also supported by all of the three phylogenetic analyses (MP, ML, BI) of the cpDNA haplotypes (see clades A–D in Figs. 3 and S1). It is interesting that *E. rituensis*, a species distributed in western Himalayas, had pure haplotype H7 of clade A, the northern China lineage. Moreover, all but four individuals of *E. saxatilis* var. *mairei*, which is sympatrically distributed with *E. likiangensis* in the Hengduan Mountains (SE QTP), harbored haplotypes (H9, H11–13) of clade D, the southern QTP lineage (Figs. 1, 3; Table 1).

### Divergence time estimation

According to the estimates by BEAST (Fig. 3; Table 3), the most recent common ancestor (MRCA) of the cpDNA haplotypes (H1–H25) detected from the 14 *Ephedra* species distributed in the QTP and adjacent regions could be dated to 27.85 Ma with the 95% highest posterior density (HPD) interval of 35.82-20.04 Ma (Fig. 3, node G). The MRCAs of the three main clades, i.e., northern China (node A), eastern QTP (node C) and southern QTP (node D), were dated to 11.35 Ma (95% HPD: 19.30-5.18 Ma), 6.84 Ma (95% HPD: 13.60-2.05 Ma) and 11.31 Ma (95% HPD: 19.92-4.55 Ma), respectively. It is interesting that the lineage D1 of clade D distributed in the east (Hengduan Mountains) also diverged early from the western lineage (Himalayas), with its MRCA dated to 5.08 Ma (95% HPD: 10.95-0.99 Ma). In general, the main clades diverged from each other in the Middle to Late Miocene.

**Table 3:** Estimates of divergence times for main lineages of cpDNA haplotypes.

Node	Median age (million years)	95% Highest posterior density intervals
A	11.35	5.18–19.30
B	5.37	0.69–12.98
C	6.84	2.05–13.60
D	11.31	4.55–19.92
D1	5.08	0.99–10.95
E	15.46	—
F	18.90	10.34–27.94
G	27.85	20.04–35.82

### Genetic differentiation within and among populations of some Ephedra species

The amount of cpDNA (*trn*T-*trn*F+*trn*S-*trn*fM) variation within and among populations was calculated using AMOVA for nine species and two varieties with a sampling size larger than two populations, separately. The three species *E. monosperma*, *E. rhytidosperma* and *E. rituensis* were excluded from the analysis due to the lack of intraspecific variation. The results showed that, for most species, genetic variation mainly occurred among populations with high *F_ST_* values (Table 4), such as *E. gerardiana* (*F*_ST_ = 0.98285), *E. saxatilis* (*F*_ST_ = 0.85572), *E. likiangensis* (*F*_ST_ = 0.92549), *E. equisetina* (*F*_ST_ = 0.77895), *E. intermedia* (*F*_ST_ = 0.86907), and *E. regeliana* (*F*_ST_ = 0.84163). Particularly, the populations of *E. przewalskii* and *E. distachya* fixed different haplotypes with *F*_ST_ = 1, although this could be partially attributed to a small sample size. In contrast, a low *F*_ST_ value (0.15317) was found in *E. minuta*, since all but two individuals of the species shared the haplotype H6. In the PERMUT analysis, no significant phylogeographic structure was detected in the species analyzed, although *N_ST_*>*G_ST_* (not significant) was observed in some species (Table 5). In addition, the Mantel test did not detect a significant correlation between genetic and geographical distances in most species (Table 4).

**Table 4:** Results of analyses of molecular variance (AMOVA) and Mantel tests for different *Ephedra* species.

Species	Source of variation	df	SS	VC	Variation (%)	Fixation index	Mantel test
*E. gerardiana*	Among populations	9	410.144	2.35820	98.29	*F_ST_* = 0.98285**	*r* = 0.462, *p* = 0.001**
	Within populations	186	7.652	0.04114	1.71		
	Total	195	417.796	2.39934			
*E. saxatilis*	Among populations	10	22.467	0.12755	85.57	*F_ST_* = 0.85572**	*r* = 0.326, *p* = 0.179
	Within populations	186	4.000	0.02151	14.43		
	Total	196	26.467	0.14906			
*E. saxatilis* var. *mairei*	Among populations	8	42.851	0.25626	54.17	*F_ST_* = 0.54167**	*r* = 0.305, *p* = 0.025
	Within populations	174	37.729	0.21683	45.83		
	Total	182	80.579	0.47309			
*E. minuta*	Among populations	9	0.371	0.00198	15.32	*F_ST_* = 0.15317	*r* = −0.094, *p* = 0.849
	Within populations	148	1.617	0.01092	84.68		
	Total	157	1.987	0.01290			
*E. likiangensis*	Among populations	15	51.852	0.21377	92.55	*F_ST_* = 0.92549**	*r* = 0.062, *p* = 0.274
	Within populations	245	4.217	0.01721	7.45		
	Total	260	56.069	0.23099			
*E. equisetina*	Among populations	9	77.265	1.28994	77.90	*F_ST_* = 0.77895**	*r* = 0.758, *p* = 0.000**
	Within populations	54	19.767	0.36605	22.10		
	Total	63	97.031	1.65599			
*E. intermedia* var. *tibetica*	Among populations	1	0.940	0.07623	16.78	*F_ST_* = 0.16777	—
	Within populations	17	6.429	0.37815	83.22		
	Total	18	7.368	0.45438			
*E. intermedia*	Among populations	9	46.562	0.57885	86.91	*F_ST_* = 0.86907**	*r* = 0.596, *p* = 0.005*
	Within populations	86	7.500	0.08721	13.09		
	Total	95	54.062	0.66606			
*E. przewalskii*	Among populations	3	20.909	1.27072	100	*F_ST_* = 1**	*r* = 0.570, *p* = 0.180
	Within populations	18	0	0	0		
	Total	21	20.909	1.27072			
*E. distachya*	Among populations	3	2.400	0.21687	0	*F_ST_* = 1*	*r* = 0.898, *p* = 0.239
	Within populations	11	0	0	0		
	Total	14	2.400	0.21687			
*E. regeliana*	Among populations	4	21.556	0.96626	84.16	*F_ST_* = 0.84163**	*r* = 0.141, *p* = 0.284
	Within populations	22	4.000	0.18182	15.84		
	Total	26	25.556	1.14808			

df, degrees of freedom; SS, sum of squares; VC, variance components; ^**^ *P*≤0.001; ^*^ *P*≤0.01; —, not calculated.

**Table 5:** Estimates of genetic diversity and population differentiation (± SE in parentheses) for *Ephedra* species.

Species	*H_S_*	*H_T_*	*G_ST_*	*N_ST_*
*E. gerardiana*	0.009 (0.0087)	0.843 (0.0541)	0.990 (0.0102)	0.985 (0.0162)^ns^
*E. saxatilis*	0.030 (0.0303)	0.498 (0.1548)	0.939 (0.0652)	0.944 (0.0599)^ns^
*E. saxatilis* var. *mairei*	0.139 (0.0795)	0.532 (0.1697)	0.739 (0.1270)	0.455 (0.1819)^ns^
*E. minuta*	0.077 (0.0663)	0.076 (0.0616)	−0.005 (NC)	−0.000 (NC)
*E. likiangensis*	0.030 (0.0243)	0.262 (0.1243)	0.887 (0.1042)	0.930 (0.0634)^ns^
*E. equisetina*	0.288 (0.1012)	0.777 (0.0726)	0.629 (0.1239)	0.630 (0.1242)^ns^
*E. intermedia* var. *tibetica*	NC	NC	NC	NC
*E. intermedia*	0.063 (0.0625)	0.625 (0.0814)	0.900 (0.0920)	0.900 (0.0920)^ns^
*E. przewalskii*	0	0.833 (0.1443)	1 (NC)	1 (NC)
*E. distachya*	0	0.500 (0.2500)	1 (NC)	1 (NC)
*E. regeliana*	0.107 (0.1067)	0.633 (0.1550)	0.832 (0.1346)	0.855 (0.1359)^ns^

*H_S_*, average genetic diversity within populations; *H_T_*, total gene diversity; *G_ST_*, interpopulation haplotype differentiation; *N_ST_*, interpopulation haplotype differentiation taking into account sequence difference; ns, *N_ST_* not significantly different from *G_ST_* (*P*>0.05); NC, not computed due to small sample size or low variation among populations.

### Mismatch distributions

Among the 14 *Ephedra* species used in the phylogeographical study, only four of them (*E. gerardiana*, *E. likiangensis*, *E. minuta*, and *E. saxatilis*) were used in the mismatch distribution analysis (Fig. S2). The other species were excluded from the analysis due to small sample size, lack of intraspecific variation, or putative historical interspecific hybridization (such as *E. saxatilis* var. *mairei*, see discussion). The results showed that the hypothesis of demographic expansion was only rejected for *E. likiangensis* (*P*_SSD_ = 0.030) (Fig. S2a). The mismatch distributions for *E. saxatilis* (*P*_SSD_ = 0.310) and *E. minuta* (*P*_SSD_ = 0.120) were unimodal (Fig. S2b, c), suggesting that the two species could have experienced recent population expansion. The two peaks in the mismatch distribution of *E. gerardiana* could be attributed to the occurrence of haplotypes from two main lineages in the species (Fig. S2d).

### Chloroplast DNA phylogeny of Ephedra

**Figure 4:**
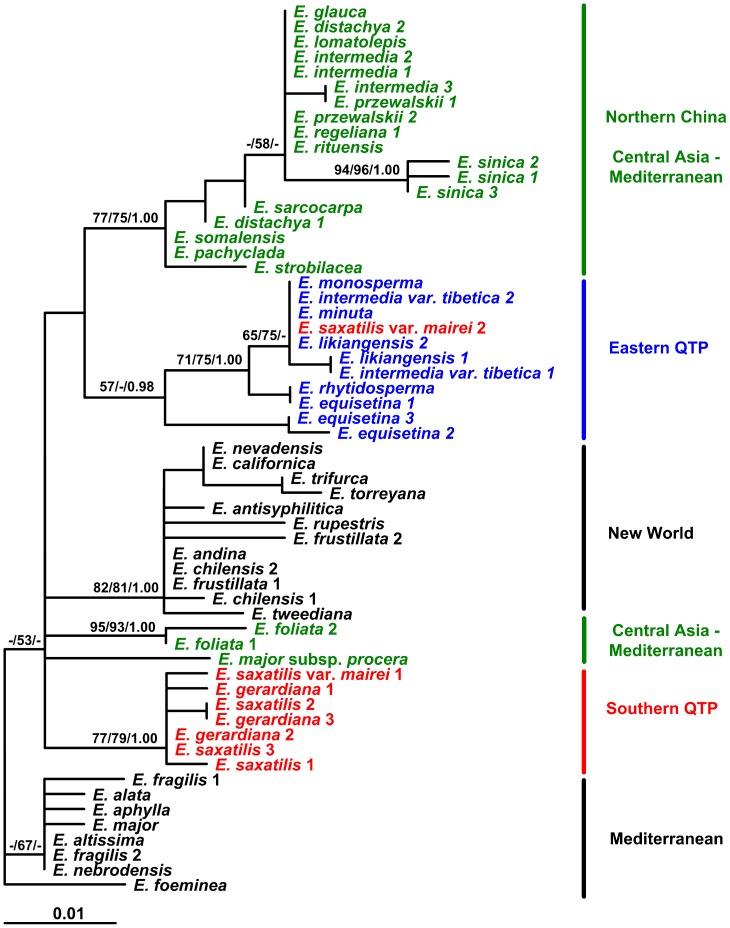
The ML tree of *Ephedra* constructed from the combined cpDNA fragments (*rbc*L, *rps*4, *rpL*16 and *trn*S-*trn*fM). Numbers above the branches are bootstrap values ≥50% for MP (left) and ML (middle) analyses, and Bayesian posterior probabilities ≥0.90 (right).

Length of the four cpDNA fragments (*rbc*L, *rps*4, *rpL*16, *trn*S-*trn*fM) was 758 bp, 443 bp, 496–516 bp and 387–458 bp, respectively. The combined alignment was 2266 bp, including 60 nucleotide substitutions and one phylogenetically informative indel (8 bp). When the Mediterranean species *E. foeminea* was used as outgroup, following Rydin *et al.* [36], the ML and BI trees generated from the combined four cpDNA fragments were generally consistent in topology, supporting five main clades that correspond well to their geographical distributions. That is, species distributed in the New World, southern QTP and eastern QTP formed monophyletic clades, respectively; Most species from the Mediterranean region clustered together; The species from northern China and Central Asia formed a clade together with a couple of species from West Asia and the Mediterranean region (*E. pachyclada*, *E. somalensis*). Like in the phylogeny of the cpDNA haplotypes (Fig. 3), *E. rituensis* from the western Himalayas was nested in the northern China clade, *E. intermedia* var. *tibetica* was located in the eastern QTP clade, and different individuals of *E. saxatilis* var. *mairei* were placed in two clades (eastern QTP and southern QTP), respectively (Fig. 4).

## Discussion

### Diversification of Ephedra in the QTP and adjacent regions was associated with uplift of the plateau and Asian aridification in the Miocene

The mechanisms underlying the development of high species diversity in the QTP are still largely unknown due to the complicated geological and climatic history of the plateau [3], [61]–[64]. Phylogeography provides a good link between population genetics and phylogenetics, and is very helpful to study speciation [65]. However, most previous phylogeographical studies of the QTP plants sampled populations of a single species and focused on the response of plant populations to the Quaternary glacial-interglacial cycles, phylogeographical structure and location of glacial refugia [18], [19], [29], [66]. In the present study, we sampled all but two of the *Ephedra* species distributed in the QTP and adjacent regions (totaling 14 species, 107 populations). Both phylogeographical analysis based on the cpDNA (*trn*T-*trn*F, *trn*S-*trn*fM) haplotypes and phylogenetic reconstruction from the combined four cpDNA fragments (*rbc*L, *rps*4, *rpL*16, *trn*S-*trn*fM) support three main lineages, i.e., eastern QTP, southern QTP, and northern China lineages of *Ephedra* (Figs. 3, 4). It is particularly interesting that the MRCA of each lineage can be dated back to the Middle or Late Miocene (Fig. 3, Table 3), a period during which the fast uplift of the QTP occurred [61], [63], [64]. Correspondingly, in the Miocene, the climate of Asia transformed from a zonal pattern to a monsoon-dominated pattern, and the aridification and desertification intensified in the Asian interior, including the QTP and northern China [2], [3], [67]. Given the high drought and/or cold tolerance of *Ephedra*, it could be inferred that the divergence of its three lineages was triggered by the Asian aridification driven by the QTP uplift. This inference is corroborated by a rich record of *Ephedra* or *Ephedra*-like fossil pollen from the late Oligocene and Miocene sediments of the QTP and neighboring areas (e.g., [58], [59], [67]). Loera *et al.* [68] also reported that the expansion of arid lands due to orogenetic and climatic changes played a role in the diversification of the North American *Ephedra* in the Late Miocene and Pliocene.

The study of Ickert-Bond *et al.* [30] indicated that *Ephedra* possibly originated in the Mediterranean region, and then dispersed into Asia, and further into the New World. If this biogeographic scenario is true, the *Ephedra* species in the QTP and neighboring regions could have evolved from a Central Asian ancestor, which possibly diverged during the QTP uplift and dispersed eastward along two routes. One was along the Himalayas, giving rise to the southern QTP lineage (Fig. 3, clade D), while the other was from Central Asia to North China, giving rise to the northern China and eastern QTP lineages (Fig. 3, clades A and C). One may argue that the eastern QTP and southern QTP lineages were once closely related, even with a sympatric distribution in the QTP, but separated afterwards due to the Quaternary climatic changes. However, this explanation is not supported by the ancient divergence between the two lineages and the sister relationship between the northern China lineage and the eastern QTP lineage (Fig. 3). Although the three haplotypes H23–H25 of *E. equisetina* are most closely related to the outgroup according to the haplotype network (Fig. 2), they show a close relationship with northern China and eastern QTP lineages in the ML trees (Figs. 3, S1). This discrepancy could be due to low resolution of the DNA markers and different algorithms of the methods. As to the distribution of *E. saxatilis* var. *mairei* (a taxon of the southern QTP lineage) in eastern QTP (Figs. 1, 3), it could be resulted from interspecific hybridization (see discussion later). It is worthy to mention that great genetic differentiation was found to have occurred between eastern QTP and southern QTP populations of single plant species (e.g., [21], [69]), but rarely between groups of congeneric species as reported in the present study.

### Low intraspecific variation and lack of strong phylogeographical structure: Implications for phylogeographical studies

Although 25 cpDNA (*trn*T-*trn*F and *trn*S-*trn*fM) haplotypes were detected in the 14 *Ephedra* species sampled from the QTP and adjacent regions, only the widely distributed *E. equisetina* has relatively rich haplotypes (7). The other species harbor 2–5 haplotypes, respectively, or lack intraspecific variation such as in *E. rhytidosperma*, *E. monosperma* and *E. rituensis* (Fig. 1; Table 1). In addition, 15 haplotypes (60%) are species-specific (Fig. 1; Table S4). Among the studied 107 populations, 89 (83%) are pure in cpDNA haplotype (Fig. 1; Table 1). Obviously, most of the *Ephedra* species have low genetic diversity (*H_T_* = 0.076–0.633; *H_S_* = 0.030–0.139) (Table 5), compared to other plants from the same region [29], such as *Pinus densata* (*H_T_* = 0.929; *H_S_* = 0.812) [22] and *Hippophae tibetana* (*H_T_* = 0.956; *H_S_* = 0.372) [69]. Although the three species *E. equisetina*, *E. gerardiana* and *E. przewalskii* have relatively high *H_T_* values (0.777–0.843), their *H_S_* values are also very low (0–0.288) (Table 5). Moreover, unlike in most previous studies that found a strong phylogeographic structure in the QTP plants [29], no significant phylogeographic structure was detected in the *Ephedra* species analyzed, although *N_ST_*>*G_ST_* (not significant) and a high *F_ST_* value were observed in some species (Tables 4, 5). This is consistent with the result of the Mantel test that genetic and geographical distances are not significantly correlated in most species (Table 4). The low intraspecific variation and lack of strong phylogeographical structure could be attributed to the prevalence of clonal reproduction, recent population expansion and a relatively recent origin of most extant species (Fig. 3), although the genus *Ephedra* could have an ancient origin [56], [70], [71]. Actually, low interspecific variation of *Ephedra* has been consistently reported in previous studies (e.g., [30], [34], [56], [68], [72]).

It seems that the level of genetic (haplotype) diversity is not obviously correlated to the wideness of distribution or ploidy level of a species. For example, among the species with a wide distribution and a good population sampling, the three species *E. equisetina* (2n = 2x = 14), *E. gerardiana* (2n = 14, 28, 56) and *E. intermedia* (2n = 4x = 28) harbor a relatively high genetic diversity, but *E. monosperma* (2n = 14, 28) lacks genetic variation (Fig. 1; Tables 1, S1). The current distribution of the QTP plants has been greatly shaped by the climatic oscillations in the late Cenozoic, especially by the Quaternary glacial-interglacial cycles, such as in *Tsuga dumosa* [20] and *Pedicularis longiflora* [19]. Despite that the mismatch distributions were only estimated for four *Ephedra* species, the results indicate different demographic histories of the congeneric species (Fig. S2). That is, unlike *E. likiangensis* from the southeastern edge of the QTP (Fig. S2a), the two species *E. minuta* and *E. saxatilis* from the eastern and southern QTP, respectively, could have experienced recent population expansion (Fig. S2b, c). This difference could be caused by different responses to the Quaternary climatic changes, although the time of population expansion needs to be further studied.

Among the 25 cpDNA haplotypes, nine (H1, H3, H5, H7, H8, H16, H19–H21) are shared by two or four species, respectively, and the haplotype H6 is even shared by eight species, suggesting that a wide sampling of species is helpful to investigate the origin of observed haplotypes and make reliable phylogeographical inference. For instance, the species *E. gerardiana* harbors five haplotypes, including H1–H3, H5, and H6 (Fig. 1; Table S4). If we only sample this species in the study, the haplotype H6 may be incorrectly considered as a newly evolved type due to its limited distribution in the species. Actually, this haplotype widely occurs in other species and is the most frequent haplotype in the eastern QTP lineage. For another example, the haplotype H7 is shared between *E. rituensis* from southern QTP and *E. regeliana* from northern China (Fig. 1; Table S4). If we only study one of the two species, it will be difficult for us to reveal the evolutionary history of this haplotype.

### Systematic positions of some Ephedra species

In the phylogenies of cpDNA haplotypes and combined four chloroplast genes, the western Himalayan *E. rituensis* is nested in the northern China lineage rather than in the southern QTP lineage, and *E. intermedia* var. *tibetica* is closely related to species of the eastern QTP lineage rather that to *E. intermedia* of the northern China lineage. Morphologically, according to our field investigation, *E. intermedia* var. *tibetica* sometimes has both straight and spirally twisted integument tubes in the same individual, which is different from *E. intermedia*. To reveal systematic positions of *E. rituensis* and *E. intermedia* var. *tibetica*, the biparentally inherited nuclear genes should be used in future studies.

In addition, there was debate about whether *E. saxatilis* var. *mairei* should be placed in *E. likiangensis* or *E. saxatilis* [31]. According to the present cpDNA analysis, *E. saxatilis* var. *mairei* is closely related to *E. saxatilis*-*E. gerardiana* rather than to *E. likiangensis*. However, our preliminary nuclear gene analysis (unpublished) seems to suggest a close relationship of *E. saxatilis* var. *mairei* to both *E. likiangensis* and *E. saxatilis*. That is, *E. saxatilis* var. *mairei* could have originated from hybridization between *E. likiangensis* and *E. saxatilis*. Actually, interspecific hybridization has been reported from the New World species [73], [74], such as the formation of ×*E. arenicola* (*E. torreyana*×*E. coryi* var. *viscida* [*E. cutleri*]) and ×*E. intermixta* (*E. torreyana*×*E. trifurca*). Moreover, *E. equisetina* harbors very different cpDNA haplotypes that are located in different clades (Fig. 3). This species has a very wide distribution in Central Asia and northern China. It would be interesting to investigate whether this species has evolved into several cryptic species given the greatly reduced morphological characters of *Ephedra*.

## Supporting Information

Figure S1 The ML tree showing evolutionary relationships of cpDNA haplotypes with H26 (*Ephedra nebrodensis*) as outgroup. Numbers above branches are bootstrap values ≥50% for MP (left) and ML (middle) analyses, and the Bayesian posterior probabilities ≥0.90 (right), respectively. Arrows denote Clades A, B, C and D.
(TIF)

Figure S2 Mismatch distributions for *Ephedra* species.
(TIF)

Table S1 Population sampling information and cpDNA (*trn*T-*trn*F+*trn*S-*trn*fM) variation of the studied *Ephedra* species.
(DOC)

Table S2 Sources of materials for phylogenetic reconstruction of *Ephedra* based on combined cpDNA.
(DOC)

Table S3 Primers used in the present study.
(DOC)

Table S4 The distribution of cpDNA haplotypes in *Ephedra* species.
(DOC)
