# Supplementary figures and images for: Phylogeographic Evidence for a Link of Species Divergence of Ephedra in the Qinghai-Tibetan Plateau and Adjacent Regions to the Miocene Asian Aridification
Source: PLoS One. 2013 Feb 13;8(2):e56243. doi: 10.1371/journal.pone.0056243 (PMC3571962; doi:10.1371/journal.pone.0056243)

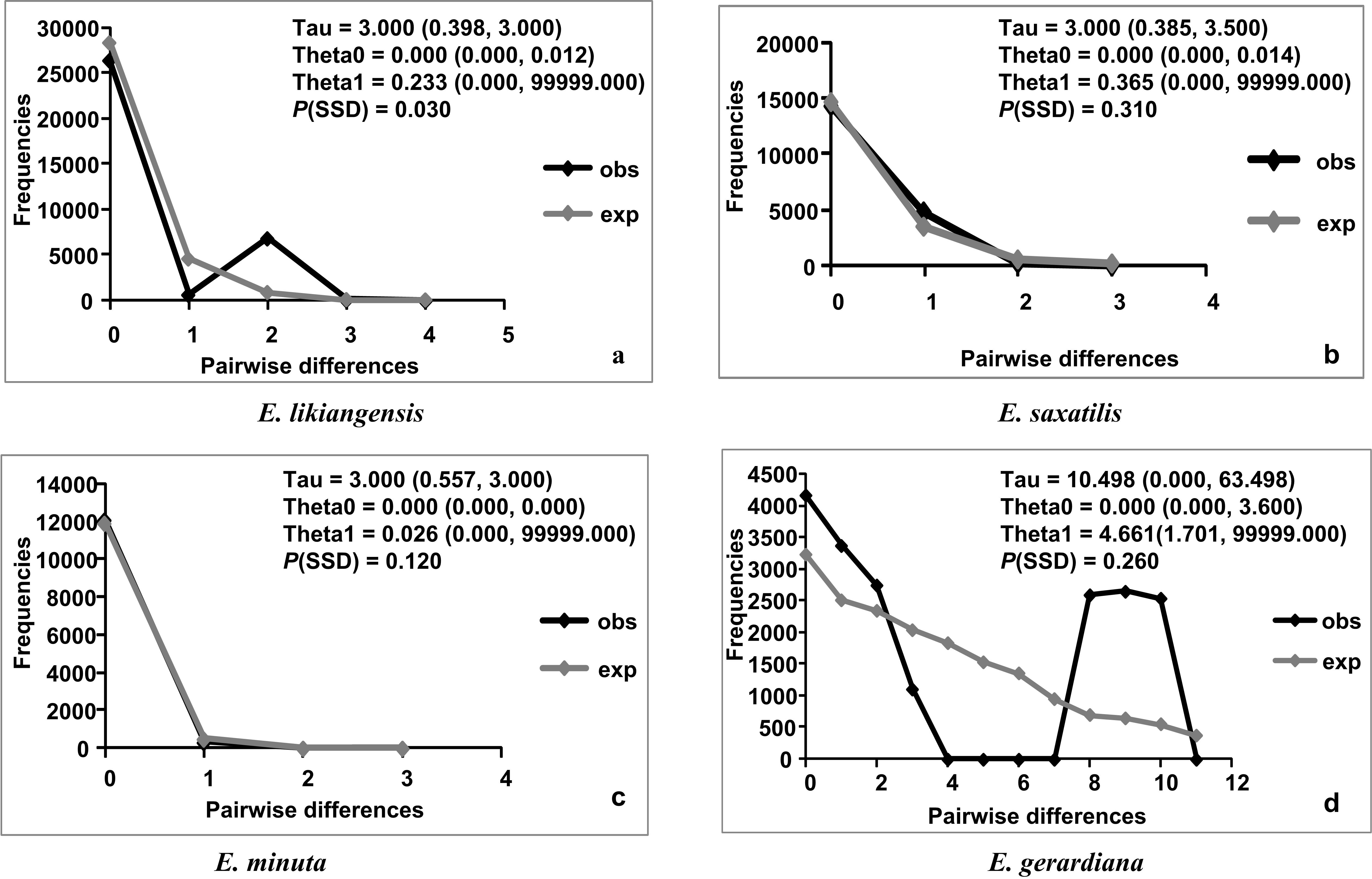

Supplement: Figure S2 — Mismatch distributions for Ephedra species. (TIF) [file pone.0056243.s002.tif]
