# Supplementary material for: Phylogeographic Evidence for a Link of Species Divergence of Ephedra in the Qinghai-Tibetan Plateau and Adjacent Regions to the Miocene Asian Aridification
Source: PLoS One. 2013 Feb 13;8(2):e56243. doi: 10.1371/journal.pone.0056243 (PMC3571962; doi:10.1371/journal.pone.0056243)
Supplement: Table S1 — Population sampling information and cpDNA (trnT-trnF+trnS-trnfM) variation of the studied Ephedra species. (DOC) [file pone.0056243.s003.doc]

**Table S1. Population sampling information and cpDNA (*trn*T-*trn*F+*trn*S-*trn*fM) variation of the studied *Ephedra* species.**

| **Species** | **Population No.** | **Sampled individuals** | **Location** | **Lat.(N)** | **Long.(E)** | **Alt.(m)** | ***S*** | ***N_h_*** | ***A*** | ***π*** | ***H_d_*** |
| --- | --- | --- | --- | --- | --- | --- | --- | --- | --- | --- | --- |
| *E*. *gerardiana* | 1 | 24 | Gar, Tibet | 32°24′ | 79°44′ | 4700 | 0 | 1 | 0.042 | 0 | 0 |
|  | 2 | 27 | Zhongba, Tibet | 29°46′ | 84°01′ | 4600 | 0 | 1 | 0.037 | 0 | 0 |
|  | 3 | 16 | Tingri, Tibet | 28°17′ | 86°48′ | 4737 | 0 | 1 | 0.063 | 0 | 0 |
|  | 4 | 24 | Rikaze, Tibet | 29°16′ | 88°51′ | 4000 | 0 | 1 | 0.042 | 0 | 0 |
|  | 5 | 23 | Damxung, Tibet | 30°27′ | 91°06′ | 4429 | 8 | 2 | 0.087 | 0.000654 | 0.0870 |
|  | 6 | 25 | Damxung, Tibet | 30°34′ | 91°12′ | 4300 | 0 | 1 | 0.040 | 0 | 0 |
|  | 7 | 3 | Cona, Tibet | 27°59′ | 91°57′ | 4400 | 0 | 1 | 0.333 | 0 | 0 |
|  | 8 | 25 | Baxoi, Tibet | 29°25′ | 96°50′ | 3970 | 0 | 1 | 0.040 | 0 | 0 |
|  | 9 | 12 | Baxoi, Tibet | 29°25′ | 96°50′ | 4068 | 0 | 1 | 0.083 | 0 | 0 |
|  | 10 | 17 | Baxoi, Tibet | 30°17′ | 97°16′ | 4150 | 0 | 1 | 0.059 | 0 | 0 |
|  | Total | 196 |  |  |  |  | 11 | 5 | 0.026 | 0.004031 | 0.7827 |
| *E*. *saxatilis* | 11 | 14 | Rutog, Tibet | 33°26′ | 79°49′ | 4400 | 0 | 1 | 0.071 | 0 | 0 |
|  | 12 | 24 | Nyalam, Tibet | 28°08′ | 85°58′ | 3900 | 0 | 1 | 0.042 | 0 | 0 |
|  | 13 | 6 | Dinggye, Tibet | 27°53′ | 87°35′ | 4200 | 0 | 1 | 0.167 | 0 | 0 |
|  | 14 | 25 | Yadong, Tibet | 27°31′ | 88°56′ | 3600 | 0 | 1 | 0.040 | 0 | 0 |
|  | 15 | 29 | Lhasa, Tibet | 29°41′ | 91°08′ | 3800 | 0 | 1 | 0.034 | 0 | 0 |
|  | 16 | 6 | Cona, Tibet | 27°55′ | 91°51′ | 4265 | 0 | 1 | 0.167 | 0 | 0 |
|  | 17 | 3 | Cona, Tibet | 27°59′ | 91°57′ | 4400 | 0 | 1 | 0.333 | 0 | 0 |
|  | 18 | 25 | Gyaca, Tibet | 29°08′ | 92°35′ | 3249 | 1 | 2 | 0.080 | 0.000311 | 0.3333 |
|  | 19 | 29 | Nang, Tibet | 29°02′ | 93°04′ | 3100 | 0 | 1 | 0.034 | 0 | 0 |
|  | 20 | 16 | Gongbo’gyamda, Tibet | 29°53′ | 93°14′ | 3441 | 0 | 1 | 0.063 | 0 | 0 |
|  | 21 | 20 | Bomi, Tibet | 29°51′ | 95°45′ | 2700 | 0 | 1 | 0.050 | 0 | 0 |
|  | Total | 197 |  |  |  |  | 3 | 4 | 0.020 | 0.000252 | 0.2576 |
| *E*. *saxatilis* var*. mairei* | 22 | 24 | Lijiang, YN | 27°03′ | 100°11′ | 3744 | 0 | 1 | 0.042 | 0 | 0 |
|  | 23 | 29 | Shangri-La, YN | 27°32′ | 99°55′ | 4100 | 0 | 1 | 0.034 | 0 | 0 |
|  | 24 | 12 | Shangri-La, YN | 27°36′ | 99°53′ | 3689 | 0 | 1 | 0.083 | 0 | 0 |
|  | 25 | 12 | Daocheng, SC | 28°26′ | 100°21′ | 3936 | 10 | 3 | 0.250 | 0.003962 | 0.7121 |
|  | 26 | 17 | Muli, SC | 28°09′ | 100°46′ | 3936 | 0 | 1 | 0.059 | 0 | 0 |
|  | 27 | 23 | Litang, SC | 29°59′ | 100°17′ | 4015 | 9 | 2 | 0.087 | 0.000736 | 0.0870 |
|  | 28 | 28 | Kangding, SC | 29°55′ | 102°00′ | 4100 | 1 | 2 | 0.071 | 0.000286 | 0.3042 |
|  | 29 | 26 | Kangding, SC | 30°02′ | 101°31′ | 3452 | 1 | 2 | 0.077 | 0.000139 | 0.1477 |
|  | 30 | 12 | Danba to Dawu, SC | 30°31′ | 101°37′ | 3540 | 0 | 1 | 0.083 | 0 | 0 |
|  | Total | 183 |  |  |  |  | 12 | 5 | 0.027 | 0.000833 | 0.4537 |
| *E*. *minuta* | 31 | 25 | Baxoi, Tibet | 29°19′ | 96°57′ | 4044 | 0 | 1 | 0.040 | 0 | 0 |
|  | 32 | 17 | Baxoi, Tibet | 29°20′ | 97°03′ | 4332 | 0 | 1 | 0.059 | 0 | 0 |
|  | 33 | 22 | Zayu, Tibet | 29°09′ | 97°10′ | 3482 | 0 | 1 | 0.045 | 0 | 0 |
|  | 34 | 3 | Zayu, Tibet | 28°45′ | 97°39′ | 4269 | 1 | 2 | 0.667 | 0.000628 | 0.6667 |
|  | 35 | 6 | Deqen, YN | 28°20′ | 99°05′ | 4182 | 0 | 1 | 0.167 | 0 | 0 |
|  | 36 | 20 | Ledu, QH | 36°15′ | 102°15′ | 3358 | 0 | 1 | 0.050 | 0 | 0 |
|  | 37 | 3 | Huzhu, QH | 36°53′ | 102°21′ | 2960 | 0 | 1 | 0.333 | 0 | 0 |
|  | 38 | 26 | Menyuan, QH | 37°40′ | 101°26′ | 3750 | 0 | 1 | 0.038 | 0 | 0 |
|  | 39 | 20 | Ulan, QH | 37°02′ | 98°39′ | 3675 | 1 | 2 | 0.100 | 0.000094 | 0.1000 |
|  | 40 | 16 | Delhi, QH | 37°29′ | 97°23′ | 3768 | 0 | 1 | 0.063 | 0 | 0 |
|  | Total | 158 |  |  |  |  | 2 | 3 | 0.019 | 0.000024 | 0.0252 |
| *E*. *likiangensis* | 41 | 22 | Zayu, Tibet | 29°09′ | 97°12′ | 3400 | 2 | 2 | 0.091 | 0.000173 | 0.0909 |
|  | 42 | 5 | Zayu, Tibet | 28°56′ | 97°25′ | 2767 | 0 | 1 | 0.200 | 0 | 0 |
|  | 43 | 24 | Deqen, YN | 28°23′ | 99°01′ | 4453 | 0 | 1 | 0.042 | 0 | 0 |
|  | 44 | 13 | Shangri-La, YN | 27°36′ | 99°53′ | 3689 | 0 | 1 | 0.077 | 0 | 0 |
|  | 45 | 25 | Shangri-La, YN | 27°46′ | 99°36′ | 3799 | 0 | 1 | 0.040 | 0 | 0 |
|  | 46 | 22 | Daocheng, SC | 28°26′ | 100°21′ | 4269 | 0 | 1 | 0.045 | 0 | 0 |
|  | 47 | 25 | Lijiang, YN | 27°04′ | 100°05′ | 3190 | 0 | 1 | 0.040 | 0 | 0 |
|  | 48 | 4 | Lijiang, YN | 27°07′ | 100°15′ | 3040 | 0 | 1 | 0.250 | 0 | 0 |
|  | 49 | 13 | Muli, SC | 28°09′ | 100°46′ | 4269 | 1 | 2 | 0.154 | 0.000365 | 0.3846 |
|  | 50 | 21 | Jinchuan, SC | 31°17′ | 101°59′ | 2900 | 0 | 1 | 0.048 | 0 | 0 |
|  | 51 | 11 | Xiaojin, SC | 30°59′ | 102°49′ | 3300 | 0 | 1 | 0.091 | 0 | 0 |
|  | 52 | 5 | Xiaojin, SC | 31°29′ | 102°29′ | 3064 | 0 | 1 | 0.200 | 0 | 0 |
|  | 53 | 23 | Barkam, SC | 31°54′ | 102°13′ | 2800 | 0 | 1 | 0.043 | 0 | 0 |
|  | 54 | 16 | Barkam, SC | 32°08′ | 102°13′ | 3460 | 0 | 1 | 0.063 | 0 | 0 |
|  | 55 | 10 | Jinchuan, SC | 31°49′ | 101°37′ | 3200 | 0 | 1 | 0.100 | 0 | 0 |
|  | 56 | 22 | Dawu, SC | 30°59′ | 101°07′ | 3007 | 0 | 1 | 0.045 | 0 | 0 |
|  | Total | 261 |  |  |  |  | 3 | 3 | 0.011 | 0.000410 | 0.2244 |
| *E*. *monosperma* | 57 | 14 | Damxung, Tibet | 30°27′ | 91°06′ | 4425 | 0 | 1 | 0.071 | 0 | 0 |
|  | 58 | 8 | Damxung, Tibet | 30°46′ | 90°52′ | 4800 | 0 | 1 | 0.125 | 0 | 0 |
|  | 59 | 16 | Golmud, QH | 35°44′ | 94°15′ | 4292 | 0 | 1 | 0.063 | 0 | 0 |
|  | 60 | 23 | Golmud, QH | 35°45′ | 94°18′ | 4287 | 0 | 1 | 0.043 | 0 | 0 |
|  | 61 | 17 | Delhi, QH | 37°21′ | 98°07′ | 3473 | 0 | 1 | 0.059 | 0 | 0 |
|  | 62 | 6 | Qinghai Lake, QH | 37°02′ | 99°44′ | 3344 | 0 | 1 | 0.167 | 0 | 0 |
|  | 63 | 6 | Minhe, QH | 36°04′ | 102°43′ | 2408 | 0 | 1 | 0.167 | 0 | 0 |
|  | 64 | 6 | Urumqi, XJ | 43°19′ | 87°12′ | 2033 | 0 | 1 | 0.167 | 0 | 0 |
|  | Total | 96 |  |  |  |  | 0 | 1 | 0.010 | 0 | 0 |
| *E*. *rhytidosperma* | 65 | 5 | Yinchuan, NX | 38°24′ | 105°55′ | 1289 | 0 | 1 | 0.200 | 0 | 0 |
|  | 66 | 6 | Yinchuan, NX | 38°36′ | 105°56′ | 1500 | 0 | 1 | 0.167 | 0 | 0 |
|  | 67 | 6 | Alxa Zuoqi, IM | 38°51′ | 105°50′ | 2150 | 0 | 1 | 0.167 | 0 | 0 |
|  | Total | 17 |  |  |  |  | 0 | 1 | 0.059 | 0 | 0 |
| *E*. *equisetina* | 68 | 6 | Yinchuan, NX | 38°44′ | 105°55′ | 1885 | 0 | 1 | 0.167 | 0 | 0 |
|  | 69 | 6 | Alxa Zuoqi, IM | 39°14′ | 106°10′ | 2000 | 0 | 1 | 0.167 | 0 | 0 |
|  | 70 | 6 | Yinchuan, NX | 38°36′ | 105°56′ | 1540 | 0 | 1 | 0.167 | 0 | 0 |
|  | 71 | 6 | Alxa Zuoqi, IM | 38°51′ | 105°52′ | 2176 | 7 | 2 | 0.333 | 0.002193 | 0.3333 |
|  | 72 | 5 | Alxa Zuoqi, IM | 38°57′ | 105°52′ | 1924 | 7 | 2 | 0.400 | 0.002632 | 0.4000 |
|  | 73 | 10 | Fuyun, XJ | 46°58′ | 89°41′ | 1243 | 0 | 1 | 0.100 | 0 | 0 |
|  | 74 | 6 | Fuhai, XJ | 47°35′ | 88°45′ | 1150 | 5 | 3 | 0.500 | 0.001566 | 0.6000 |
|  | 75 | 7 | Altay, XJ | 47°49′ | 88°10′ | 1291 | 0 | 1 | 0.143 | 0 | 0 |
|  | 76 | 6 | Hoboksar, XJ | 46°33′ | 86°01′ | 860 | 5 | 2 | 0.333 | 0.001566 | 0.3333 |
|  | 77 | 6 | Shawan, XJ | 43°56′ | 85°51′ | 1305 | 0 | 1 | 0.167 | 0 | 0 |
|  | Total | 64 |  |  |  |  | 12 | 7 | 0.109 | 0.002895 | 0.6984 |
| *E*. *glauca* | 78 | 5 | Urumqi, XJ | 43°37′ | 87°57′ | 1450 | 3 | 2 | 0.400 | 0.001119 | 0.4000 |
| *E*. *przewalskii* | 79 | 5 | Delhi, QH | 37°16′ | 97°10′ | 2910 | 0 | 1 | 0.200 | 0 | 0 |
|  | 80 | 5 | Urumqi, XJ | 43°33′ | 87°53′ | 1128 | 0 | 1 | 0.200 | 0 | 0 |
|  | 81 | 6 | Hoboksar, XJ | 46°33′ | 86°04′ | 941 | 0 | 1 | 0.167 | 0 | 0 |
|  | 82 | 6 | Burqin, XJ | 47°41′ | 86°50′ | 493 | 0 | 1 | 0.167 | 0 | 0 |
|  | Total | 22 |  |  |  |  | 5 | 3 | 0.136 | 0.001846 | 0.6277 |
| *E*. *intermedia* | 83 | 5 | Songpan, SC | 32°19′ | 103°43′ | 3000 | 0 | 1 | 0.200 | 0 | 0 |
|  | 84 | 5 | Minhe, QH | 35°53′ | 102°48′ | 1866 | 0 | 1 | 0.200 | 0 | 0 |
|  | 85 | 27 | Qinghai Lake, QH | 36°38′ | 100°26′ | 3207 | 0 | 1 | 0.037 | 0 | 0 |
|  | 86 | 22 | Qinghai Lake, QH | 36°33′ | 100°28′ | 3565 | 0 | 1 | 0.045 | 0 | 0 |
|  | 87 | 16 | Qilian, QH | 38°13′ | 100°12′ | 2950 | 2 | 2 | 0.125 | 0.000927 | 0.5000 |
|  | 88 | 2 | Urumqi, XJ | 43°47′ | 87°33′ | 999 | 0 | 1 | 0.500 | 0 | 0 |
|  | 89 | 5 | Manas, XJ | 44°14′ | 86°20′ | 478 | 0 | 1 | 0.200 | 0 | 0 |
|  | 90 | 6 | Shawan, XJ | 43°57′ | 85°46′ | 1205 | 0 | 1 | 0.167 | 0 | 0 |
|  | 91 | 6 | Shawan, XJ | 44°09′ | 85°37′ | 828 | 0 | 1 | 0.167 | 0 | 0 |
|  | 92 | 2 | Hoboksar, XJ | 46°32′ | 86°03′ | 861 | 0 | 1 | 0.500 | 0 | 0 |
|  | Total | 96 |  |  |  |  | 6 | 5 | 0.052 | 0.001055 | 0.5037 |
| *E*. *intermedia* var*. tibetica* | 93 | 14 | Baxoi to Bomi, Tibet | 29°40′ | 96°12′ | 3170 | 2 | 2 | 0.143 | 0.000939 | 0.4945 |
|  | 94 | 5 | Zayu, Tibet | 29°09′ | 97°12′ | 3275 | 0 | 1 | 0.200 | 0 | 0 |
|  | Total | 19 |  |  |  |  | 2 | 2 | 0.105 | 0.000778 | 0.4094 |
| *E*. *rituensis* | 95 | 12 | Rutog, Tibet | 33°26′ | 79°49′ | 4400 | 0 | 1 | 0.083 | 0 | 0 |
|  | 96 | 30 | Burang, Tibet | 30°11′ | 81°15′ | 3700 | 0 | 1 | 0.033 | 0 | 0 |
|  | 97 | 24 | Gyirong, Tibet | 28°40′ | 85°16′ | 3900 | 0 | 1 | 0.042 | 0 | 0 |
|  | Total | 66 |  |  |  |  | 0 | 1 | 0.015 | 0 | 0 |
| *E*. *distachya* | 98 | 3 | Fuhai, XJ | 47°34′ | 88°42′ | 1213 | 0 | 1 | 0.333 | 0 | 0 |
|  | 99 | 5 | Burqin, XJ | 47°40′ | 86°48′ | 543 | 0 | 1 | 0.200 | 0 | 0 |
|  | 100 | 4 | Hoboksar, XJ | 46°32′ | 86°03′ | 861 | 0 | 1 | 0.250 | 0 | 0 |
|  | 101 | 3 | Jeminay, XJ | 47°41′ | 85°55′ | 669 | 0 | 1 | 0.333 | 0 | 0 |
|  | Total | 15 |  |  |  |  | 1 | 2 | 0.133 | 0.000318 | 0.3429 |
| *E*. *sinica* | 102 | 13 | Hexigten Qi, IM | 43°34′ | 117°10′ | 1350 | 0 | 1 | 0.077 | 0 | 0 |
| *E*. *regeliana* | 103 | 6 | Shawan, XJ | 43°56′ | 85°25′ | 1306 | 0 | 1 | 0.167 | 0 | 0 |
|  | 104 | 6 | Burqin to Altay, XJ | 47°40′ | 86°48′ | 543 | 3 | 2 | 0.333 | 0.001483 | 0.5333 |
|  | 105 | 5 | Urumqi, XJ | 43°39′ | 87°39′ | 1130 | 0 | 1 | 0.200 | 0 | 0 |
|  | 106 | 5 | Shawan, XJ | 43°57′ | 85°46′ | 1159 | 0 | 1 | 0.200 | 0 | 0 |
|  | 107 | 5 | Urumqi, XJ | 43°25′ | 87°13′ | 1723 | 0 | 1 | 0.200 | 0 | 0 |
|  | Total | 27 |  |  |  |  | 4 | 3 | 0.111 | 0.001822 | 0.5698 |
| **All species** | **Total** | **1435** |  |  |  |  | **28** | **25** | **0.017** | **0.004854** | **0.7867** |
| *E. nebrodensis* |  | 1 | Mallorca, Spain |  |  |  |  |  |  |  |  |

Abbreviations: YN, Yunnan; SC, Sichuan; QH, Qinghai; NX, Ningxia; IM, Inner Mongolia; XJ, Xinjiang; Lat., latitude; Long., longitude; Alt., altitude; *S*, number of segregating sites; *N_h_*, number of haplotypes; *A*, haplotype richness; *π*, nucleotide diversity; *H*_d_, haplotype diversity.
