# Supplementary material for: Phylogeographic Evidence for a Link of Species Divergence of Ephedra in the Qinghai-Tibetan Plateau and Adjacent Regions to the Miocene Asian Aridification
Source: PLoS One. 2013 Feb 13;8(2):e56243. doi: 10.1371/journal.pone.0056243 (PMC3571962; doi:10.1371/journal.pone.0056243)
Supplement: Table S2 — Sources of materials for phylogenetic reconstruction of Ephedra based on combined cpDNA. (DOC) [file pone.0056243.s004.doc]

**Table S2.** Sources of materials for phylogenetic reconstruction of *Ephedra* based on combined cpDNA.

| **Taxa** | **Vouchers** | **Sources** | **GenBank accession numbers** | | | | **Distribution** |
| --- | --- | --- | --- | --- | --- | --- | --- |
|  |  |  | ***rbc*L** | ***rps*4** | ***rpL*16** | ***trn*S-*trn*fM** |  |
| *E. alata* | C-303 (S) | Algeria | AY755805 | AY755851 | FJ958074 | FJ958162 | Mediterranean |
| *E. altissima* | C- 628 (S) | Algeria - Morocco | AY755803 | AY755849 | FJ958072 | FJ958160 | North Africa |
|  | C-7688 (S) | Tunisia | AY755804 | AY755850 | FJ958073 | FJ958161 |  |
| *E. andina* | 10140 (K) | Kew Garden (cult.) | AY755782 | AY755821 | FJ958045 | FJ958128 | South America |
| *E. antisyphilitica* | 451 (S) | Oklahoma, USA | AY755789 | AY755834 | FJ958057 | FJ958145 | North America |
| *E. aphylla* | C-7791 (S) | Libya | AY755802 | AY755848 | FJ958071 | FJ958159 | Mediterranean |
| *E. californica* | 68.154 (O) | University of Oslo Botanical Garden (cult.) | AY056569 | AY755827 | FJ958050 | FJ958135 | North America |
| *E. chilensis* 1 | 7780 (E) | Chile | AY755786 | AY755831 | FJ958054 | FJ958142 | South America |
| *E. chilensis* 2 | 49.0542 (UC) | Chile | AY755799 | AY755844 | FJ958067 | FJ958155 |  |
| *E. distachya* 1 | S04-481 (S) | 68-226 (cult.) | AY755793 | AY755838 | FJ958061 | FJ958149 | Europe-Asia |
| *E. distachya* 2 | WMM191 (PE) | FH, XJ, China | KC222975 | KC223067 | KC223021 | KC223113 |  |
|  | WMM085 (PE) | Jeminay, XJ, China | KC222976 | KC223068 | KC223022 | KC223114 |  |
| *E. equisetina* 1 | WMM082 (PE) | Alxa Zuoqi, IM, China | KC222977 | KC223069 | KC223023 | KC223115 | Central-East Asia |
| *E. equisetina* 2 | WMM023 (PE) | Shawan, XJ, China | KC222978 | KC223070 | KC223024 | KC223116 |  |
| *E. equisetina* 3 | WMM174 (PE) | Altay, XJ, China | KC222979 | KC223071 | KC223025 | KC223117 |  |
| *E. foeminea* | 26068 (UPS) | Israel | FJ958029 | AY591478 | FJ958084 | FJ958172 | Mediterranean |
|  | 2098 (S) | Turkey | AY755808 | AY755855 | FJ958078 | FJ958166 |  |
| *E. foliata* 1 | C-7808 (S) | Iran | AY755806 | AY755853 | FJ958076 | FJ958164 | Mediterranean |
|  | 10745 (UPS) | Somalia | FJ958032 | FJ958111 | FJ958087 | FJ958175 | Central Asia |
| *E. foliata* 2 | 92019A (UPS) | Saudi Arabia | FJ958031 | FJ958110 | FJ958086 | FJ958174 |  |
| *E. fragilis* 1 | 5412 (UPS) | Morocco | FJ958034 | FJ958115 | FJ958090 | FJ958176 | Mediterranean |
| *E. fragilis* 2 | 179-01 (S) | Morocco | FJ958037 | FJ958119 | FJ958095 | FJ958181 |  |
| *E. frustillata* 1 | 10218 (K) | Kew Garden (cult.) | AY056564 | AY755825 | FJ958048 | FJ958131 | South America |
| *E. frustillata* 2 | 08 (S) | Patagonia, Chile | AY056570 | AY755820 | FJ958044 | FJ958127 |  |
| *E. gerardiana* 1 | 10141 (K) | Kew Garden (cult.) | AY056560 | AY755822 | FJ958046 | FJ958129 | Central-East Asia |
| *E. gerardiana* 2 | MacPherson s.n. (E) | Sikkim, India | AY755785 | AY755830 | FJ958053 | FJ958141 |  |
|  | CYZ20060926 (PE) | Rikaze, Tibet, China | KC222980 | KC223072 | KC223026 | KC223118 |  |
| *E. gerardiana* 3 | CYZ20060906 (PE) | Gar, Tibet, China | KC222981 | KC223073 | KC223027 | KC223119 |  |
| *E. glauca* | WMM012 (PE) | Urumqi, XJ, China | KC222982 | KC223074 | KC223028 | KC223120 | China |
| *E. intermedia* 1 | 04.483(S) | Mt. Tianshan (cult.) | AY755790 | AY755835 | FJ958058 | FJ958146 | Central-East Asia |
| *E. intermedia* 2 | 03.925 (S) | Mt. Tianshan (cult.) | AY056566 | AY755818 | FJ958042 | FJ958125 |  |
|  | WMM001 (PE) | Urumqi, XJ, China | KC222983 | KC223075 | KC223029 | KC223121 |  |
|  | WMM037 (PE) | Shawan, XJ, China | KC222984 | KC223076 | KC223030 | KC223122 |  |
| *E. intermedia* 3 | CYZ20100805 (PE) | Songpan, SC, China | KC222985 | KC223077 | KC223031 | KC223123 |  |
|  | YFS20100824 (PE) | Qilian, QH, China | KC222986 | KC223078 | KC223032 | KC223124 |  |
| *E. intermedia* var. *tibetica* 1 | YFS20090621 (PE) | Baxoi to Bomi, Tibet, China | KC222987 | KC223079 | KC223033 | KC223125 | China |
| *E. intermedia* var. *tibetica* 2 | YFS20090621 (PE) | Baxoi to Bomi,Tibet, China | KC222988 | KC223080 | KC223034 | KC223126 |  |
|  | WMM428 (PE) | Zayu, Tibet, China | KC222989 | KC223081 | KC223035 | KC223127 |  |
| *E. likiangensis* 1 | 03.926 (S) | Denver Botanic Gardens (Cult.) | AY755780 | AY755816 | FJ958040 | FJ958123 | China |
|  | QAL0620003 (PE) | Lijiang, YN, China | KC222990 | KC223082 | KC223036 | KC223128 |  |
| *E. likiangensis* 2 | 94.0389 (UC) | YN, China | AY755798 | AY755843 | FJ958066 | FJ958154 |  |
|  | WMM413 (PE) | Zayu, Tibet, China | KC222991 | KC223083 | KC223037 | KC223129 |  |
|  | CYZ20100809 (PE) | Jinchuan, SC, China | KC222992 | KC223084 | KC223038 | KC223130 |  |
| *E. lomatolepis* | Baitulin (UPS) | Kazakhstan | FJ958028 | FJ958108 | FJ958083 | FJ958171 | Central-East Asia |
| *E. major* | 03.164 (S) | Spain | AY755809 | AY755856 | FJ958079 | FJ958167 | Mediterranean –Central Asia  central Asia |
| *E. major* subsp*. procera* | C-242 (S) | Algeria | FJ958035 | FJ958117 | FJ958092 | FJ958178 | Mediterranean –Central Asia  central Asia |
| *E. minuta* | YFS20090711 (PE) | Baxoi, Tibet, China | KC222993 | KC223085 | KC223039 | KC223131 | China |
|  | LB876 (PE) | Zayu, Tibet, China | KC222994 | KC223086 | KC223040 | KC223132 |  |
|  | QAL0730001 (PE) | Ledu, QH, China | KC222995 | KC223087 | KC223041 | KC223133 |  |
|  | WMM261 (PE) | Delhi, QH, China | KC222996 | KC223088 | KC223042 | KC223134 |  |
| *E. monosperma* | QAL0809002 (PE) | Damxung, Tibet, China | KC222997 | KC223089 | KC223043 | KC223135 | Central-East Asia |
|  | QAL0804002 (PE) | Delhi, QH, China | KC222998 | KC223090 | KC223044 | KC223136 |  |
|  | WMM043 (PE) | Urumqi, XJ, China | KC222999 | KC223091 | KC223045 | KC223137 |  |
| *E. nebrodensis* | WXQ2139 (PE) | Mallorca, Spain | KC223000 | KC223092 | KC223046 | KC223138 | Mediterranean |
| *E. nevadensis* | 66.1033 (UC) | California, USA (cult.) | AY755796 | AY755841 | FJ958064 | FJ958152 | North America |
| *E. pachyclada* | C-7844 (S) | Sinai, Egypt | AY755810 | AY755857 | FJ958080 | FJ958168 | West Asia |
| *E. przewalskii* 1 | WMM251 (PE) | Delhi, QH, China | KC223001 | KC223093 | KC223047 | KC223139 | Central-East Asia |
| *E. przewalskii* 2 | WMM007 (PE) | Urumqi, XJ, China | KC223002 | KC223094 | KC223048 | KC223140 |  |
|  | WMM107 (PE) | Burqin, XJ, China | KC223003 | KC223095 | KC223049 | KC223141 |  |
| *E. regeliana* | WMM033 (PE) | Shawan, XJ, China | KC223004 | KC223096 | KC223050 | KC223142 | Central-East Asia |
|  | WMM014 (PE) | Urumqi, XJ, China | KC223005 | KC223097 | KC223051 | KC223143 |  |
|  | WMM047 (PE) | Urumqi, XJ, China | KC223006 | KC223098 | KC223052 | KC223144 |  |
| *E. rhytidosperma* | WMM062 (PE) | Yinchuan, NX, China | KC223007 | KC223099 | KC223053 | KC223145 | China |
|  | WMM054 (PE) | Yinchuan, NX, China | KC223008 | KC223100 | KC223054 | KC223146 |  |
|  | WMM072 (PE) | Alxa Zuoqi, IM, China | KC223009 | KC223101 | KC223055 | KC223147 |  |
| *E. rituensis* | CYZ20060904 (PE) | Rutog, Tibet, China | KC223010 | KC223102 | KC223056 | KC223148 | China |
|  | CYZ20060909 (PE) | Burang, Tibet, China | KC223011 | KC223103 | KC223057 | KC223149 |  |
|  | CYZ20060921 (PE) | Gyirong, Tibet, China | KC223012 | KC223104 | KC223058 | KC223150 |  |
| *E. rupestris* | 87.1368 (UC) | Ecuador (cult.) | AY755797 | AY755842 | FJ958065 | FJ958153 | South America |
| *E. sarcocarpa* | 2.786 (S) | Iran | AY492045 | FJ958118 | FJ958093 | FJ958179 | Central Asia |
| *E. saxatilis* 1 | C-218 (S) | Tibet, China | FJ958038 | AY591491 | FJ958098 | FJ958184 | East-South Asia |
| *E. saxatilis* 2 | YFS20060904 (PE) | Rutog, Tibet, China | KC223013 | KC223105 | KC223059 | KC223151 |  |
| *E. saxatilis* 3 | CYZ20060827 (PE) | Lhasa, Tibet, China | KC223014 | KC223106 | KC223060 | KC223152 |  |
|  | WMM380 (PE) | Gyaca, Tibet, China | KC223015 | KC223107 | KC223061 | KC223153 |  |
|  | CYZ20061021 (PE) | Bomi, Tibet, China | KC223016 | KC223108 | KC223062 | KC223154 |  |
| *E. saxatilis* var*. mairei* 1 | QAL0623010 (PE) | Shangri-La, YN, China | KC223017 | KC223109 | KC223063 | KC223155 | China |
|  | CYZ20100816 (PE) | Kangding, SC, China | KC223018 | KC223110 | KC223064 | KC223156 |  |
| *E. saxatilis* var*. mairei* 2 | WMM473 (PE) | Daocheng, SC, China | KC223019 | KC223111 | KC223065 | KC223157 |  |
| *E. sinica* 1 | EtOH (S) | Hebei, China | AY056565 | AY755826 | FJ958049 | FJ958134 | East Asia |
| *E. sinica* 2 | 10143 ( K) | (cult.) | AY056562 | AY755824 | FJ958047 | FJ958130 |  |
| *E. sinica* 3 | WXQ20091012 (PE) | Hexigten Qi, IM, China | KC223020 | KC223112 | KC223066 | KC223158 |  |
| *E. somalensis* | 10925A (UPS) | Somalia | FJ958027 | AY591444 | FJ958081 | FJ958169 | Horn of Africa |
| *E. strobilacea* | 2703 (S) | Iran | FJ958036 | AY591448 | FJ958094 | FJ958180 | Central Asia |
| *E. trifurca* | 04630447 (MO) | Arizona, USA (cult.) | AY755794 | AY755839 | FJ958062 | FJ958150 | North America |
| *E. torreyana* | 04.487 (S) | New Mexico, USA | AY755791 | AY755836 | FJ958059 | FJ958147 | North America |
| *E. tweediana* | 66.0742 (UC) | Argentina | AY755800 | AY755845 | FJ958068 | FJ958156 | South America |

Abbreviations: YN, Yunnan; SC, Sichuan; QH, Qinghai; NX, Ningxia; IM, Inner Mongolia; XJ, Xinjiang.
