# Supplementary material for: Phylogeographic Evidence for a Link of Species Divergence of Ephedra in the Qinghai-Tibetan Plateau and Adjacent Regions to the Miocene Asian Aridification
Source: PLoS One. 2013 Feb 13;8(2):e56243. doi: 10.1371/journal.pone.0056243 (PMC3571962; doi:10.1371/journal.pone.0056243)
Supplement: Table S3 — Primers used in the present study. (DOC) [file pone.0056243.s005.doc]

**Table S3. Primers used in the present study.**

| **Region** | **Primer (5’-3’)** | | **Amplified sequence length (bp)** | **Reference** |
| --- | --- | --- | --- | --- |
| *trn*T*-trn*F | *trn*T | CATTACAAATGCGATGCTCT | 668 | [1] |
|  | *trn*F | ATTTGAACTGGTGACACGAG |  |  |
|  |  |  |  |  |
| *trn*S*-trn*fM | *trn*S | GAGAGAGAGGGATTCGAACC | 387-458 | [2] |
|  | *trn*fM | CATAACCTTGAGGTCACGGG |  |  |
|  |  |  |  |  |
| *rbc*L | *rbc*L5’ | ATGTCACCACAAACAGAGAC | 758 | [3] |
|  | *rbc*L3’ | TCAAATTCAAACTTGATTTCTTTCCA |  | [4] |
|  |  |  |  |  |
| *rpL*16 | *rpL*16_F71 | GCTATGCTTAGTGTGTGACTCGTTG | 496-516 | [5] |
|  | *rpL*16_R1516 | CCCTTCATTCTTCCTCTATGTTG |  |  |
|  |  |  |  |  |
| *rps*4 | *rps*4Fb | CGATCTTCTCGACCCTGGTGG | 443 | [6] |
|  | *rps*4Rb | CCGTCGAGAATAATATTCTAT |  |  |

1. Taberlet P, Gielly L, Pautou G, Bouvet J (1991) Universal primers for amplification of three non-coding regions of chloroplast DNA. Plant Mol Biol 17: 1105–1109.
2. Demesure B, Sodzi N, Petit RJ (1995) A set of universal primers for ampliﬁcation of polymorphic non-coding regions of mitochondrial and chloroplast DNA in plants. Mol Ecol 4: 129–131.
3. Zurawski G, Clegg MT (1987) Evolution of higher plant chloroplast DNA-encoded genes: implications for structure-function and phylogenetic studies. Annu Rev Plant Biol 38: 391–418.
4. Wikström N, Kenrick P (1997) Phylogeny of *Lycopodiaceae* (Lycopsida) and the relationship of *Phylloglossum drumondii* Kunze based on *rbc*L sequence data. Int J Plant Sci 158: 862–871.
5. Small RL, Ryburn JA, Cronn RC, Seelanan T, Wendel JF (1998) The tortoise and the hare: choosing between noncoding plastome and nuclear ADH sequences for phylogeny reconstruction in a recently diverged plant group. Amer J Bot 85: 1301–1315.
6. Rydin C, Pedersen KR, Friis EM (2004) On the evolutionary history of *Ephedra*: Cretaceous fossils and extant molecules. Proc Natl Acad Sci USA 101: 16571–16576.
