# Supplementary material for: Phylogeographic Evidence for a Link of Species Divergence of Ephedra in the Qinghai-Tibetan Plateau and Adjacent Regions to the Miocene Asian Aridification
Source: PLoS One. 2013 Feb 13;8(2):e56243. doi: 10.1371/journal.pone.0056243 (PMC3571962; doi:10.1371/journal.pone.0056243)
Supplement: Table S4 — The distribution of cpDNA haplotypes in Ephedra species. (DOC) [file pone.0056243.s006.doc]

**Table S4.** The distribution of cpDNA haplotypes in *Ephedra* species.

| **Haplotype**  **Species** | **H1** | **H2** | **H3** | **H4** | **H5** | **H6** | **H7** | **H8** | **H9** | **H10** | **H11** | **H12** | **H13** | **H14** | **H15** | **H16** | **H17** | **H18** | **H19** | **H20** | **H21** | **H22** | **H23** | **H24** | **H25** |
| --- | --- | --- | --- | --- | --- | --- | --- | --- | --- | --- | --- | --- | --- | --- | --- | --- | --- | --- | --- | --- | --- | --- | --- | --- | --- |
| *E. gerardiana* | 24 | 46 | 24 |  | 47 | 55 |  |  |  |  |  |  |  |  |  |  |  |  |  |  |  |  |  |  |  |
| *E. saxatilis* | 14 |  | 5 | 9 | 169 |  |  |  |  |  |  |  |  |  |  |  |  |  |  |  |  |  |  |  |  |
| *E. saxatilis* var*. mairei* |  |  |  |  |  | 4 |  |  | 132 |  | 17 | 25 | 5 |  |  |  |  |  |  |  |  |  |  |  |  |
| *E. minuta* |  |  |  |  |  | 156 |  |  |  | 1 |  |  |  | 1 |  |  |  |  |  |  |  |  |  |  |  |
| *E. likiangensis* |  |  |  |  |  | 228 |  | 30 |  |  |  |  |  |  | 3 |  |  |  |  |  |  |  |  |  |  |
| *E. monosperma* |  |  |  |  |  | 96 |  |  |  |  |  |  |  |  |  |  |  |  |  |  |  |  |  |  |  |
| *E. rhytidosperma* |  |  |  |  |  | 17 |  |  |  |  |  |  |  |  |  |  |  |  |  |  |  |  |  |  |  |
| *E. glauca* |  |  |  |  |  |  |  |  |  |  |  |  |  |  |  | 1 |  |  |  |  | 4 |  |  |  |  |
| *E. equisetina* |  |  |  |  |  | 27 |  |  |  |  |  |  |  |  |  | 2 |  |  |  | 5 | 1 |  | 22 | 6 | 1 |
| *E. intermedia* var*. tibetica* |  |  |  |  |  | 14 |  | 5 |  |  |  |  |  |  |  |  |  |  |  |  |  |  |  |  |  |
| *E. rituensis* |  |  |  |  |  |  | 66 |  |  |  |  |  |  |  |  |  |  |  |  |  |  |  |  |  |  |
| *E. intermedia* |  |  |  |  |  |  |  |  |  |  |  |  |  |  |  | 65 | 10 | 17 | 2 | 2 |  |  |  |  |  |
| *E. przewalskii* |  |  |  |  |  |  |  |  |  |  |  |  |  |  |  | 5 |  |  |  | 12 | 5 |  |  |  |  |
| *E. sinica* |  |  |  |  |  |  |  |  |  |  |  |  |  |  |  |  |  |  |  |  |  | 13 |  |  |  |
| *E. distachya* |  |  |  |  |  |  |  |  |  |  |  |  |  |  |  |  |  |  |  | 12 | 3 |  |  |  |  |
| *E. regeliana* |  |  |  |  |  |  | 2 |  |  |  |  |  |  |  |  |  |  |  | 15 | 10 |  |  |  |  |  |

The haplotypes shared by two or more species are shown in red. Values in the table are number of individuals.
